# Asymmetric Monomethine Cyanine Dyes with Hydrophobic Functionalities for Fluorescent Intercalator Displacement Assay

**DOI:** 10.3390/molecules29010114

**Published:** 2023-12-23

**Authors:** Sonia Ilieva, Nadezhda Bozova, Miroslav Rangelov, Nadezhda Todorova, Aleksey Vasilev, Diana Cheshmedzhieva

**Affiliations:** 1 Faculty of Chemistry and Pharmacy, Sofia University “St. Kliment Ohridski”, 1 J. Bourchier Ave., 1164 Sofia, Bulgaria; silieva@chem.uni-sofia.bg (S.I.); nadegdabozova@abv.bg (N.B.); 2 Institute of Organic Chemistry with Centre of Phytochemistry, Bulgarian Academy of Sciences, 1113 Sofia, Bulgaria; miroslav.rangelov@orgchm.bas.bg; 3 Institute of Biodiversity and Ecosystem Research, Bulgarian Academy of Sciences, 1113 Sofia, Bulgaria; nadeshdahr@gmail.com; 4 Institute of Polymers, Bulgarian Academy of Sciences, Akad. G. Bonchev St., Bl 103A, 1113 Sofia, Bulgaria

**Keywords:** asymmetric monomethine cyanine dyes, RNA, DNA, fluorescence titration, biomolecular probes, DFT, TDDFT

## Abstract

A new green procedure has been applied for the synthesis and purification of asymmetric monomethine cyanine dyes. The photophysical properties of the newly synthesized compounds have been examined by combined application of spectroscopic and theoretical methods. The structural characteristics of the molecules and dimer formation were characterized by quantum chemical computation and juxtaposed to the aggregachromism in UV/Vis spectra. The applicability of the dyes as fluorogenic nucleic acid probes has been proven by fluorescence titration, and their binding constants have been calculated. The mode of ligand–dsDNA/RNA interaction was rationalized by means of CD spectroscopy, molecular docking analysis, and fluorescent intercalator displacement experiments.

## 1. Introduction

The development of technology goes hand in hand with the emergence and improvement of new bioanalytical techniques [1]. This, in turn, necessitates the creation of new and more perfect equipment and new bioanalytical reagents with which it can function precisely. More concretely, contemporary methods such as flow cytometry, fluorescence in situ hybridization, fluorescence spectroscopy, and PCR analysis cannot exist without fluorogenic dyes to report the presence of target biomolecules in the analyzed samples [1]. Almost all types of organic dyes are used as visualizing reagents, but the most widely used are cyanine dyes. This is due to the fact that these dyes do not have their own fluorescence in molecular solutions but acquire one in contact with certain bio-analytes. They usually target a defined type of biomolecule, making them quite selective without requiring a covalent bond between them and the bio-object of interest. The easy and cheap methods of synthesis as well as the possibility of easy functionalization and modification of the structures can be pointed out as advantages of cyanine dyes over other classes of dyes. Small changes in the molecular structure of the dyes can lead to significant changes in their photophysical properties. In this way, due to the listed advantages of cyanine dyes, maximum results can be achieved with minimal efforts. Very often, the change of only one atom or one functional group leads to drastic changes in the photophysical properties of the dyes. Furthermore, changing the side groups in the same type of chromophores can result in targeting different cellular organelles. Moreover, the growing interest in these dyes and their ever-wider application in biomedical analytical methods necessitates the development of new green procedures for their preparation and purification. So-called “green chemistry” involves a new approach to the synthesis, processing, and application of chemical substances, thereby reducing the harmful impact on human health and preventing environmental pollution. It is presented as a set of twelve principles proposed by Anastas and Warner [2,3,4]. These principles include instructions for professional chemists in the creation of new compounds, new syntheses, and new technological processes and procedures. The first principle describes the idea of green chemistry [2,3,4] protecting the environment from pollution. The remaining principles focus on such issues as atom economy, non-toxicity, solvents, energy consumption, use of starting materials from renewable sources, and the decomposition of chemical products into simple non-toxic substances that are compatible with the environment [2,3,4]. As we already mentioned, in the last few years, the application of fluorogenic dyes derived from thiazole orange (TO) or acridine orange as visualization reagents in a number of biomedical analyzes such as PCR, flow cytometry, fluorescence, confocal microscopy, etc. greatly expanded in scope. The COVID-19 pandemic has established PCR analysis as the dominant method for fast and accurate detection of infectious pathogens, suggesting an increase in the production of such fluorogenic dyes. For this reason, the development and use of environmentally friendly synthetic methods, consistent with the principles of green chemistry, is of significant importance regarding the scale-up synthesis of dyes like TO and its analogs. In the present work, we develop such environmentally friendly procedures.

In this regard, the present paper aims to describe new environmentally friendly approaches for the synthesis of monomethine cyanine dyes. Furthermore, we would like to demonstrate the applicability of thus-synthesized cyanine dyes as fluorogenic biomolecular probes for nucleic acid analysis and especially for fluorescent intercalator displacement (FID) assay.

## 2. Results

### 2.1. Synthesis of Intermediates and Dyes

**Scheme 1:**
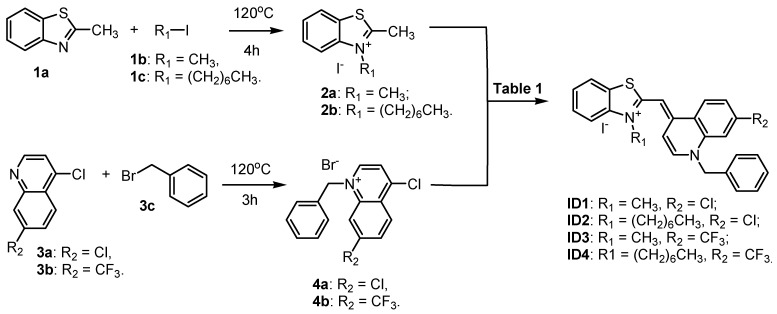
Synthetic strategy for the preparation of dyes ID1–ID4.

In our previous work, [5] we demonstrated a sustainable approach for the synthesis of quaternary nitrogen containing heterocyclic compounds. Synthesis takes place in a short time in a melt of the two components. Thus, by solvent-free melting of 2-methylbenzothiazole (**1a**) and the corresponding alkyl iodide **1b** or **1c**, we synthesized the quaternary benzothiazole salts **2a** and **2b** (Scheme 1).

A similar strategy was used in the reaction of quaternization between 4,7-dichloroquinoline **3a** and 4-chloro-7-(trifluoromethyl)quinoline **3b** and benzyl bromide **3c** to obtain dye intermediates 1-benzyl-4,7-dichloroquinolin-1-ium bromide 4a and 1-benzyl-4-chloro-7-(trifluoromethyl)quinolin-1-ium bromide **4b** (Scheme 1). The synthesis was described by our group earlier [6]. The condensation reaction between the CH-acidic methyl group of the benzothiazole ring activated by the quaternary nitrogen atoms in the chromophore and the 4-chloro-quinolinium quaternary derivatives was carried out in ethanol or water as solvents and in the presence of sodium hydrogen carbonate as a basic reagent (experimental part). In the first procedure A, the reaction proceeded after grounding the reactants 2,3-dimethylbenzo[d]thiazol-3-ium iodide (**2a**) or 3-heptyl-2-methylbenzo[d]thiazol-3-ium iodide (**2b**) and 1-benzyl-4,7-dichloroquinolin-1-ium bromide (**4a**) or 1-benzyl-4-chloro-7-(trifluoromethyl)quinolin-1-ium bromide (**4b**) in a mortar in the presence of an excess of sodium hydrogen carbonate as a basic reagent. The reaction occurred in the presence of small amounts of ethanol, and its progress was followed by TLC monitoring. In the second procedure B, we used a mechanochemical approach as well and a double molar excess of sodium bicarbonate and water as a green solvent. Generally, the reaction yields are a bit higher in the presence of ethanol compared to water. All selected reaction conditions allowed a significant shortening of the reaction procedures to one hour of reaction time (experimental part). Additionally, the applied synthetic conditions ensure that the reaction will proceed in a slurry and require quite small amounts of so called “green solvents” like ethanol and water. We can point out the lack of foul-smelling pollution of methyl mercaptan that happens during Brooker’s method [7] as an advantage of the reaction.

Dyes ID1 and ID3 were previously prepared by the classical method [6]. To the best of our knowledge, dyes ID2 and ID4 are new compounds that have not been published so far. Their chemical structures were proven by NMR, UV-VIS, and fluorescence spectroscopy.

### 2.2. Photophysical Properties of Dyes and Their Complexes with dsDNA and RNA

The main characteristics of the absorption spectra of the dyes and their complexes with dsDNA and RNA are listed in Table 1. The absorption spectra of the dyes in TE buffer show two overlapping absorption bands in the interval 490–520 nm that can be attributed to the dye monomers and associates. The higher wavelength and higher intensity band (512–520 nm) are characteristic of the dyes’ monomers. Chlorine containing analogues ID1 and ID2 show higher molar absorptivity. Self-aggregation of asymmetric cyanine dyes in a water solution is a well-known phenomenon [8]. The cyanine dyes can form different aggregates with varying structures depending on the dye itself. The shorter wavelength maxima (489–498 nm) that can be attributed to the formation of dimers is blue-shifted with 18–23 nm (Table 1).

**Figure 1:**
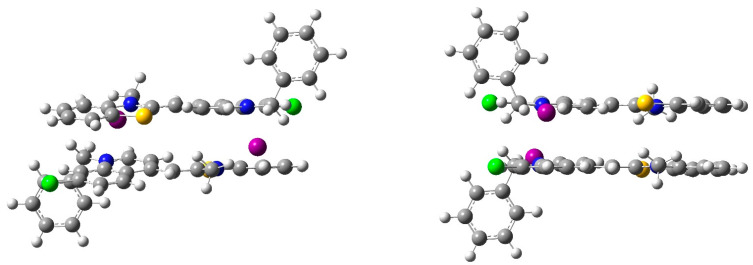
PCM/M06-2X/6-31G(d,p) optimized geometry of the two most stable π-stacked dimers of the dye ID1.

Density functional theory (DFT) and time dependent DFT (TDDFT) methods were employed for calculating the photophysical properties of the newly synthesized compounds and their aggregates. Cis and trans conformers with respect to the C=C double bond are possible. It has been unequivocally proven that trans conformation is more stable, and only trans conformers exist in ambient conditions [9,10]. The geometry of the π-stacked dimers of the dyes studied was optimized at M06-2X/6-31G(d,p) in a water solvent (SDD basis set used for the iodide counterions) [11,12]. The spatial orientation of the two most stable π-stacked dimers for ID1 is shown in Figure 1. The computed Gibbs free energy difference between these two dimers is 1.19 kcal/mol. The predicted vertical absorption values for the two most stable dimers for each of the dyes are listed in Table 2. It can be seen that the predicted absorption maxima of the dimers (474–493 nm) are blue-shifted according to the calculated absorption for the respective monomers (501–512 nm). The obtained theoretical results are in agreement with the experimental observations (Table 2).

**Figure 2:**
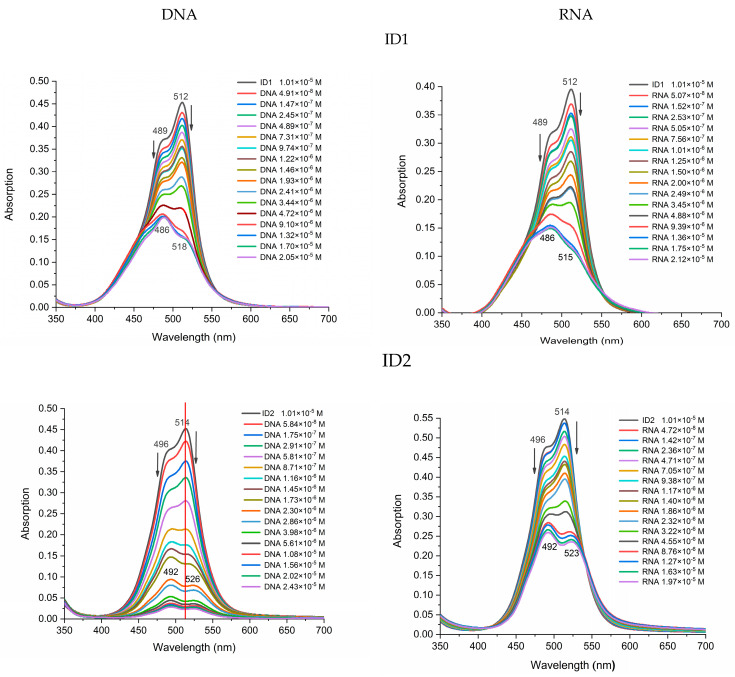
UV/Vis absorption spectra of chlorine containing dyes ID1 and ID2 pure in TE buffer and evolution of spectra upon addition of DNA and RNA.

The changes in the absorption spectra of the dyes upon the addition of definite quantities of DNA and RNA are shown in Figure 2 and Figure S8. The proportional decrease in the intensity of the long-wave maximum is evidence of the monomer–nucleic acid interaction. It can be seen from Figure 2 that the shorter wavelength maxima also decrease in intensity with the addition of NA. The hypochromic and bathochromic shifts of the absorption maxima of the dye-NA complexes observed during nucleic acid titration are evidence of the dye-NA interaction. The bathochromic shift of the longer wavelength maximum—4 nm and 14 nm for ID1 and ID2, respectively—is shown in Figure 2.

**Figure 3:**
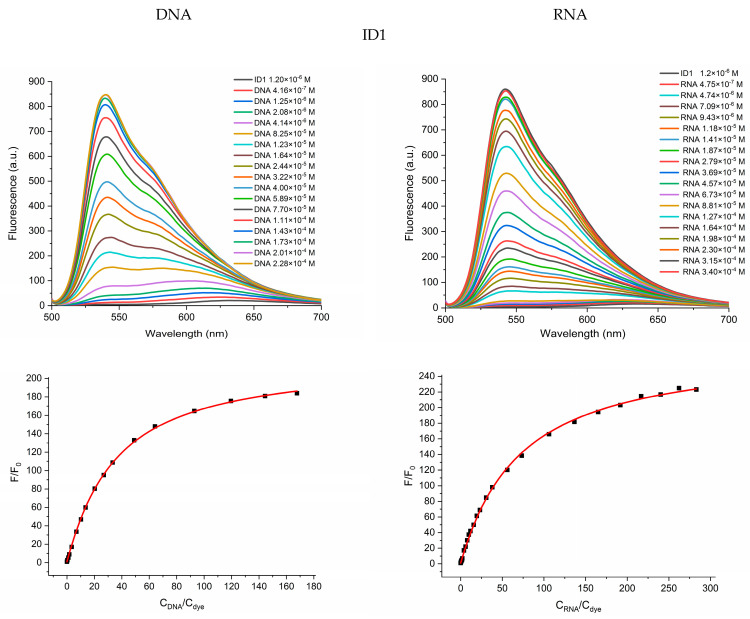

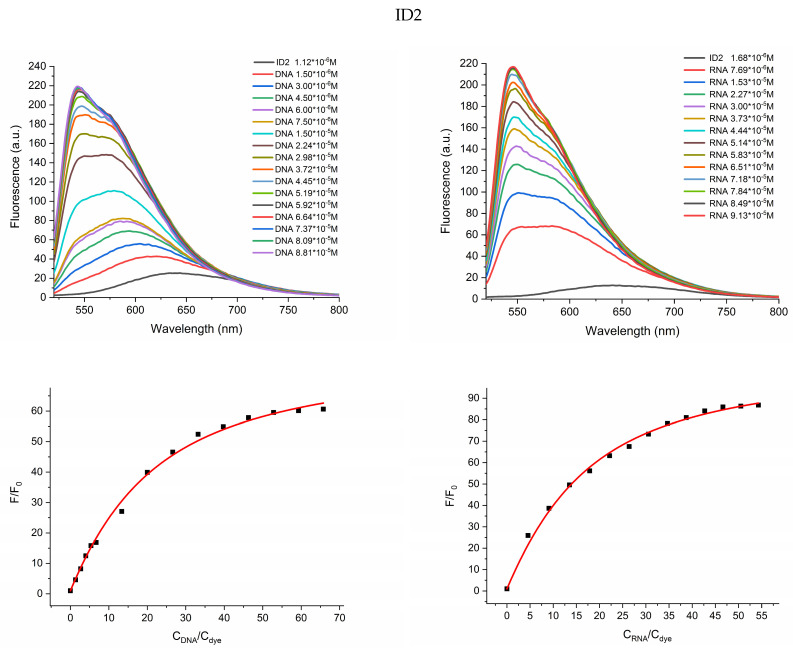
Fluorescence spectra of chlorine-containing dyes ID1 and ID2 neat in TE buffer and evolution of spectra upon addition of DNA and RNA.

The dye–nucleic acid interactions were also examined by fluorescence spectroscopy. The changes in the fluorescence spectra of the dyes upon the addition of definite quantities of DNA and RNA are shown in Table 3, Figure 3 and Figure S9. All dyes possess insignificant intrinsic fluorescence in TE buffer, while a considerable increase in fluorescence intensity is observed upon binding to dsDNA or RNA (Table 3). The methyl-substituted dyes ID1 and ID3 demonstrate higher sensitivity than alkyl analogs ID2 and ID4. The elongation of the hydrocarbon chain of the substituent in the benzothiazole ring leads to a decrease in the fluorescence response upon binding of the dyes to nucleic acids—ID2 and ID4 compared to ID1 and ID3, respectively. The most significant fluorescence response upon binding to nucleic acids is observed for ID1—an increase in the fluorescence intensity of 183 and 223 times for the complexes with DNA and RNA, respectively (Table 3). The dye ID1 shows higher sensitivity to RNA in comparison to dsDNA. The latter conclusion is consistent with the previously demonstrated dependence that the chlorine-substituted dyes exhibit higher sensitivity to RNA in comparison to dsDNA [13].

Fluorescence titration of each of the dyes with dsDNA and RNA was performed to determine their binding constants (Table 4). In this fluorescence titration experiment, the nucleic acid (dsDNA or RNA) solution of a particular concentration is gradually added to the dye solution and incubated for 5 min before fluorescence measurement. Titration data were analyzed according to the site-independent model by nonlinear fitting to Equation (1). The parameters A and Q in Equation (1) were found via the Levenberg-Marquardt fitting routine in the Origin 2023 software [14], at n = 0.1 for each dye.

(1) $$F/F_{0}=1+\frac{\left(Q-1\right)}{2}*\left[A+n*x+1-\sqrt{{\left(A+n*x\right)}^{2}-4*n*x}\right]$$

In this equation, F_0_ is the fluorescence intensity of the neat dye, and F_max_ is the fluorescence intensity upon saturation with NA and the parameters A = 1/(K_b_ ∗ C_dye_), Q = F_max_/F_0_, and n = C_dye_/C_DNA_ [15].

The values of the binding constants K_b_ suggest strong interactions with the nucleic acids and a partial intercalative mode of interaction [5,16]. It can be seen from the last two columns of Table 4 that ID2 binds strongly to both NAs. On the other side from the data in Table 3, it is clear that ID1 exhibits the highest sensitivity upon excitation with both dsDNA and RNA. The ID1 demonstrates the greatest increase in the fluorescence intensity of 183- and 223-fold for the complexes with DNA and RNA, respectively. A study of specific oligonucleotides to determine the specific interactions with base pairs can be envisaged as a future prospect. This will be a base for directed synthesis and design of new DNA probes.

**Figure 4:**
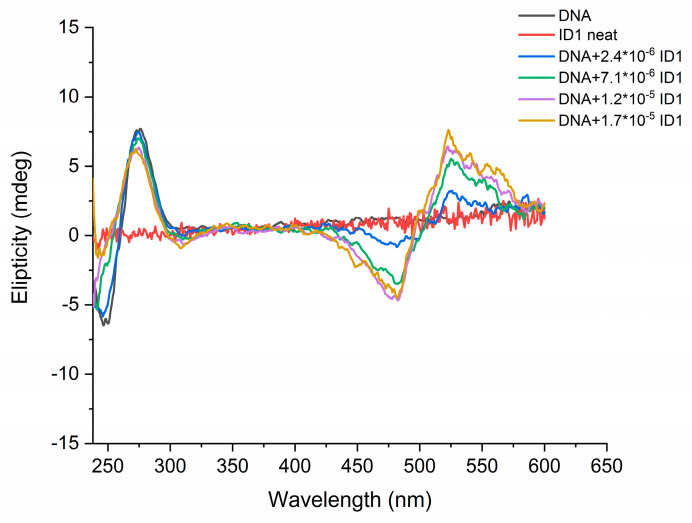
CD spectra of DNA neat and upon addition of ID1 in TE buffer.

Circular dichroism (CD) measurements of ID1 dye were performed in TE buffer and in the presence of DNA in order to gain insight into the possible mode of dye–DNA interaction. The CD spectrum of dsDNA shows negative and positive bands at 247 nm and 275 nm (indicating a B-DNA conformation). The dye itself does not have a CD signal in the visible region. After increasing amounts of ID1 are added to the DNA solution, an induced exciton CD signal is observed. The signal has a bisignate shape, with one positive and one negative band located on either side of the absorption maximum of the free ligand (Figure 4). According to Garbett et al. [17], the ICD signal is sensitive to whether the ligand binds as a monomer or dimer, and such an exciton CD is indicative for the formation of dimers, or higher order complexes, in either a groove-binding or an external stacking binding mode, although there are exceptions reported in the literature [18,19].

Fluorescent intercalator displacement (FID) assay, developed by Borger et al., is a fast and sensitive analytical method for determining the type and affinity of ligand binding to DNA [20,21,22]. The basis of the method is the competitive displacement of ethidium bromide (EB) or thiazole orange (TO) from the DNA-EB or DNA-TO complexes by a new DNA binding molecule that results in quenching the fluorescence of the initial complexes DNA-EB/DNA-TO. This method is applicable to both high-performance screening and quantitative titration for determining binding constants. Thus, the FID method is applicable to investigate the mechanism of the interaction between a ligand and DNA in the development of new drugs targeting DNA. Liu et al. [23] have published a study on the mechanism of the interaction between DNA and rare earth cation complexes with dibromo-8-hydroxy quinoline. The authors have proven the type of binding using several analytical methods—circular dichroism, viscosimetry, gel electrophoresis, and UV-VIS spectroscopy, as well as through competitive intercalation of EB and metal complexes of dibromo-8-hydroxy quinolone. A slightly modified FID assay is applied in the present work.

**Figure 5:**
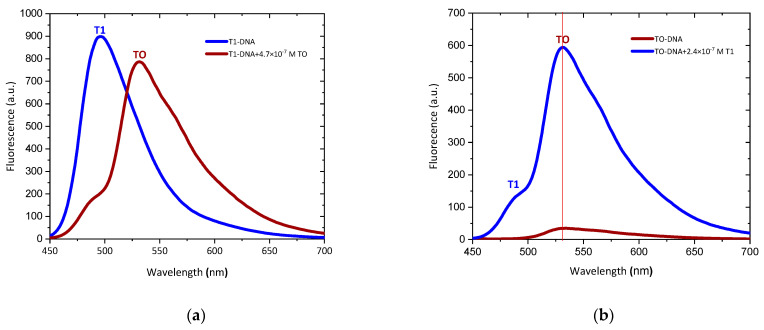
Fluorescent intercalator displacement assay for the dyes TO and T1: (**a**) Fluorescence of the T1-DNA complex (blue line, C_T1_ = 2.4 × 10^−7^ M) and the change upon addition of TO (maroon line); (**b**) Fluorescence of the TO-DNA complex (maroon line) and the change upon addition of T1 (blue line, C_T1_ = 2.4 × 10^−7^ M).

There are some strong inconveniences when working with EB or TO in FID assay. Ethidium bromide is mutagenic and toxic. TO binds very strongly to DNA and can hardly be displaced by other dyes or potential drugs. An additional obstacle in our case is that the emission maximum of TO is very close to the emission maxima of the studied dyes. Hence the aim is to find a new reference intercalator that does not bind very strongly to DNA and that can be displaced from its DNA complexes. The use of this relatively weak intercalator would be to act as a reference in testing other dyes or drugs for their binding mode to DNA. The fluorescent intercalator displacement between TO and T1 dyes has been examined. TO is an established strong intercalator. T1 dye has also been proven as an intercalator using CD spectroscopy and fluorescence titration in our previous work [24]. The change in fluorescence of the T1-DNA complex upon addition of TO is shown in Figure 5. The T1-DNA complex has an emission maximum at λ_max_ 495 nm (excitation at 430 nm). As demonstrated, the T1 dye shows a very strong increase in fluorescence when bonded to DNA. When TO (4.7 × 10^−7^ M) is added to the complex, the intensity of the band at 495 nm decreases significantly, and a new band appears at 554 nm, characteristic for the TO-DNA complex. Thiazole orange is an intercalator, and this behavior (Figure 5a) indicates that TO displaces T1 and intercalates in its place. When T1 is added to a TO-DNA complex (Figure 5b), only a shoulder at 495 nm appears, testifying that T1 is a weaker intercalator and cannot displace TO from its complexes. Therefore, the dye T1 can be applied as a reference intercalator in FID assay.

**Figure 6:**
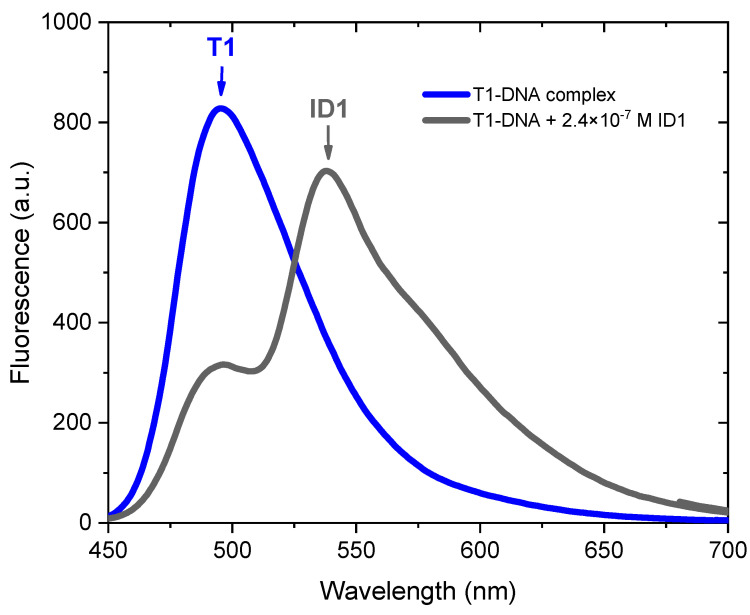
Fluorescence of T1-DNA complex (blue line, C_T1_ = 2.4 × 10^−7^ M) and the change upon addition of ID1 (gray line, C_ID1_ = 2.4 × 10^−7^ M).

**Figure 7:**
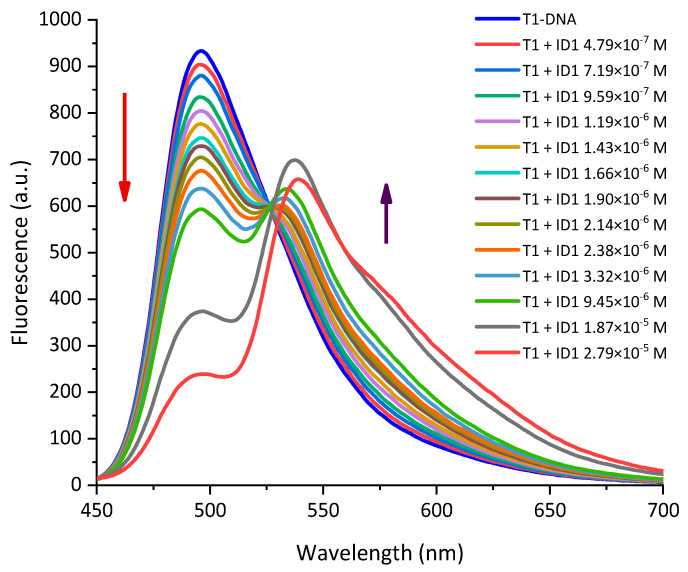
Fluorescence titration of T1-DNA complex (C_T1_ = 2.4 × 10^−7^ M) with ID1.

T1 is a suitable reference intercalator for the dyes ID1–ID4 since the T1-DNA emission maximum differs substantially from the emission maxima of the DNA complexes of the studied dyes. The change in fluorescence of the T1-DNA complex upon addition of ID1 is shown in Figure 6. When ID1 (2.4 × 10^−7^ M) is added to the T1-DNA complex, the band at 495 nm decreases significantly, and a new band at 542 nm appears, characteristic for the ID1-DNA complex (see Table 3). This change is due to the displacement of T1 by ID1. A titration utilizing the fluorescent intercalator displacement was conducted by adding aliquots of ID1 dye to the T1-DNA complex (Figure 7). The change in the fluorescence at each step was measured after 5 min equilibration time. It can be seen from Figure 7 that the 495 nm fluorescence signal decreases upon addition of dye ID1, and the 542 nm maximum increases, thus showing the formation of the ID1-DNA complex. The isosbestic point corresponds to the equilibrium of the two complexes.

### 2.3. Molecular Docking

Docking studies were performed to shed light on the favorite mode of interaction of the asymmetric monomethine cyanine dyes with double-strand DNA (dsDNA) and double-strand RNA (dsRNA). Three major potential modes of interaction were explored: with minor or major grooves and by intercalating.

Ligand preparation for the docking procedure includes proper protonation, followed by conformational search, where possible conformations of the ligands are generated by a low mode molecular dynamic generator using an AMBER12EHT force field and a generalized born solvent model as implemented in MOE 2.14 software [25]. Only conformations within a 10 kcal/mol frame from the lowest energy one were used in the docking procedure.

The docking of ligands in the major and minor grooves of dsDNA is quite clear as a procedure, while intercalators are puzzling as they position themselves between bases, thus generating de novo holes in dsNA depending on the size of the ligand. PDB structures of dsDNA with intercalated ligands of different sizes were employed for generating the most suitable pockets. A series of 3D XRD structures were sorted out and employed as docking templates. The selected PDB codes of the dsDNA structures were as follows: 108D, 185D, 1DNE, 1XRW, 1Z3F, 2DES. Excluding only 1DNE, other complexes represent dsDNA with an intercalated ligand. The 1DNE was used as a kind of control for the possible formation of complexes alongside the DNA grooves. Short (50 ns) molecular dynamic (MD) simulations with NAMD software were performed to relax XRD structures and to condition them in a solution. AMBER12EHT force field and explicit water molecules in periodic boundary conditions at 310 K and 1 atm were used.

The preparation of dsRNA models was based on the modification of the same dsDNA XRD structures to dsRNA constructs, followed by 250 ns molecular dynamic simulations with the same simulation parameters as in dsDNA. The longer simulation time is due to the structural differences between dsRNA and dsDNA. The structures from MD simulations were modified by removing the respective ligands. These structures were further applied for positioning ligands in the grooves for the docking procedure. The natural process of intercalation results in a cavity in the dsDNA/dsRNA, with the best possible size for the intercalating molecule, i.e., there is not a stable, well-defined rigid pocket in the NA structure, according to which a ligand would undergo conformational changes to fit inside. Therefore, a pocket was created de novo, and the double-stranded nucleic acid structure underwent drastic conformational changes during the intercalation process. For this reason, we have chosen the induced fit docking methodology for all of the steps, used to elucidate interaction energies between our ligands and nucleic acids. This methodology would allow optimal mutual fitting of the ligand and its pocket in the dsDNA/dsRNA.

As a first step in the docking procedure, two different placement algorithms were used, and the poses returned by them were combined further in the analysis. The first placement algorithm was the triangle matching algorithm, with up to 1,000,000 poses inside the cavities, and the second was the AlphaPMI algorithm [25]. All produced poses were scored by the London dG scoring function, and the best 100 nonduplicating poses were used in an additional induced fit minimization step with the AMBER12EHT force field and the generalized born solvent model. The only constraint in the course of minimization was that the dsNA atoms, further than 6 Å from the ligand atoms, as well as the backbone atoms, were considered unmovable. The final scoring of the poses was performed by dG, calculated with the GBVI/WSA scoring function [26].

The analysis of the data obtained revealed that despite the relatively long structure of the ligands, their length is not enough to allow interaction with dsDNA or dsRNA in a bidentate mode. The best interactions are between the ligands and 1XRW. However, when considering the interaction with dsDNA and dsRNA grooves, the length of our intercalating species is enough for the interaction with the 3′ or 5′ ends of nucleic acid models.

The second step in the docking procedure concerns the unification of the receptor used to obtain comparable interaction energies. The 1XRW was used as a receptor structure. Elongated models of 1XRW were prepared—up to 16 bp (base pairs) of dsDNA and dsRNA—to overcome the interaction of the ligands with 3′ and 5′ ends. The resultant structures were subjected to new (100 ns) MD simulations for relaxation.

A third docking study was performed to find the preferred NA base pairs for ligand intercalation. Synthetic models, containing different base pairs to surround the ligand, were used for the docking. The dsDNA and dsRNA models consist of six base pairs, where a gap of 7.5 Å forms between the first three bp and the second three. These can be G-C_G-C_G-C, A-C_A-C_A-C, or A-U_A-U_A-U, thus forming all possible neighboring combinations around the intercalating ligands. DsDNA receptors were prepared in the B form of nucleic acid. dsRNA was prepared in the A form, as these preferences are well known and also confirmed by the performed MD simulations. The gap itself is formed by elongating one of the ester bonds, which connect the phosphate to the ribose ring. The two-step induced fit docking protocol was used. At the first step, the triangle match algorithm was used for placement of the ligands, and the London dG scoring function was used for scoring the poses. The best 100 poses for each ligand at every pocket were further fully optimized without any restriction during the minimization, using the AMBER12EHT force field and the generalized born solvent model. The minimized complexes were scored by the GBVI/WSA scoring function.

It was found that the studied ligands (dyes ID1-ID4) cannot interact with the major grooves of dsDNA and dsRNA, because in all cases, at the optimization stage of the induced fit docking procedure, part of the ligands penetrate the double-stranded nucleic acid, thus forming low energy intercalation complexes. For this reason, all subsequent data represent interactions with the minor groove of the double-stranded nucleic acid and are marked “in the groove” in the text. The interaction energies for the dye-NA complexes are given in Table 5. All of the studied ligands prefer to interact as intercalators with both dsRNA and ds DNA, although the energy differences are not large. The dyes ID2 and ID4, possessing a long alkyl substituent in the benzothiazole ring, form the best dsDNA complexes with lower interaction energies. ID4 shows the lowest interaction energies with dsRNA.

**Figure 8:**
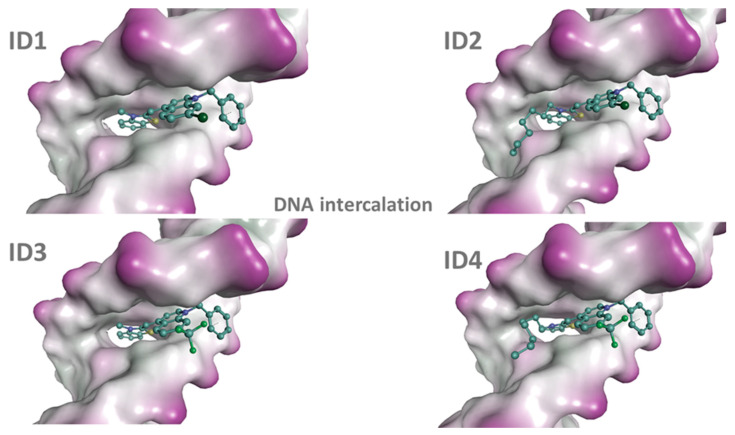
The best poses for the intercalation complexes of the dyes ID–ID4 to dsDNA (1XRW). The purple color represents the hydrophilic areas in DNA.

All ligands interacted better with the minor groove of dsDNA than with the minor groove of dsRNA. Such an effect is understandable, bearing in mind that the dsDNA minor groove is deeper than the same groove in dsRNA. In the case of longer ligands, dyes ID2 and ID4, the preference is to place the long alkyl substituent along the groove, while in the other two cases (dyes ID1 and ID3), the methyl group is oriented toward the solution (Figure 8). However, there is no such dependence for complexes with dsRNA.

The favored ligand position in dsDNA intercalation complexes is stacked between the nucleobases and the benzothiazole ring, while the benzyl group and the long alkyl substituent (ID2, ID4) are placed in the minor groove. Additional interactions between the minor groove and the long alkyl substituent lead to lower energy complexes of ID2 and ID4 with dsDNA.

In the case of dsRNA intercalation, the preferred orientation is stacked between the nucleobases and the quinoline ring, while the benzyl group interacts with the minor groove. The benzothiazole ring is placed on the side of the major groove, and for ID2 and ID4 ligands, the long alkyl substituent interacts with the major groove, thus leading to better interaction energies of these ligands.

To shed light on the preferred nucleobases for the ligands’ intercalation, we performed a series of docking/minimization studies. The results obtained demonstrate that in all cases, our ligands prefer to intercalate between two A-T pairs in dsDNA. In the case of dsRNA, the best ligand, ID4, as well as ID2, also prefer two A-U pairs, ID3 prefers two G-C pairs, and the ligand ID1 prefers A-U bp from one side and G-C bp from the other side.

The ICD indicates a binding mode by groove binding and aggregation in the groove. It is clear that these hydrophobic dyes readily form dimers. However, the FID analysis suggests that the ID1 dye is an intercalator because it displaces the intercalating dye T1 from its complexes with DNA and quenches its fluorescence (Figure 6 and Figure 7). The energy differences from molecular docking are not large enough to exclude the minor groove binding mode of interaction. The combination of methods points to multiple binding modes. Presumably, at low ligand/nucleotide binding ratios (C_dye_/C_DNA_ = 0.007), intercalation is the preferred mode of interaction, as evidenced from the FID assay experiments. At higher binding ratios (C_dye_/C_DNA_ = 0.02), groove-binding aggregates are formed.

## 3. Materials and Methods

All solvents used in the present work are HPLC grade and commercially available. The starting materials **1a**, **1b**, **1c**, **3a**, **3b**, and **3c** are commercially available, and they were used as supplied. Melting points of ID1–ID4 were determined on a Büchi MP B-545 apparatus and are uncorrected. *NMR spectra* (^1^H-, ^13^C-NMR) were obtained on a Bruker Avance II + NMR spectrometer operating at 500 MHz for ^1^H- and 125 MHz for ^13^C-NMR in DMSO-d_6_ as a solvent. The chemical shifts are given in ppm (δ) using tetramethylsilane (TMS) as an internal standard. UV-VIS spectra were measured on a Shimadzu UV-1800 spectrophotometer. Calibration curves for estimating the molar absorption coefficients are given in Figures S10–S13. The fluorescence spectra were obtained on a PerkinElmer LS45 fluorescence spectrophotometer. Fresh stock solutions (1 mM) were prepared in DMSO and further diluted with TE buffer (Tris-HCl 10 mM pH 8.0; EDTA 1 mM, pH 8.0) to 1.10^−5^ M for the UV–VIS spectra and to 1.10^−6^ M for the fluorescence spectra. The salmon sperm dsDNA (CAS 68938-01-2) and baker’s yeast RNA (CAS 63231-63-0) were purchased from Merck (Darmstadt, Germany) (Sigma-Aldrich, St. Louis, MO, USA) and used as received. The stock concentrations of DNA and RNA were 1 mg/mL stock. The purity of the nucleic acids was checked by the absorption spectrum and the ratio at 260 and 280 nm. The operating concentration of the DNA and RNA working solutions was defined spectroscopically following molar extinction coefficients at the wavelengths indicated: dsDNA—6600 M^−1^, RNA—7800 L.mol^−1^.cm^−1^. [27].

The fluorescence spectra of cyanine dyes were recorded in the range 220–700 nm. All UV-VIS and fluorimetric titrations were carried out by keeping the dye concentration constant while varying the DNA and RNA concentrations (10 mM Tris-HCl, 1 mM EDTA buffer, pH 8 working solutions). After the addition of the aliquots of NA, the solution was equilibrated for 5 min before recording the spectra.

CD spectra were recorded on a JASCO J815 spectrophotometer. CD experiments were performed by adding portions of ID1 dye into the solution of the dsDNA (c = 1 × 10^−4^ M).

### 3.1. Procedure A

1 mmol 2,3-dimethylbenzo[d]thiazol-3-ium iodide (**2a**) or 3-heptyl-2-methylbenzo[d]thiazol-3-ium iodide (**2b**) and 1.1 mmol 1-benzyl-4,7-dichloroquinolin-1-ium bromide (**4a**) or 1-benzyl-4-chloro-7-(trifluoromethyl)quinolin-1-ium bromide (**4b**) and 2.2 mmol sodium hydrogen carbonate were ground together in a mortar in the presence of 200 μml of ethanol. The reaction mixture was grounded vigorously for 30 min. An additional 5 mL of ethanol was added, and the mixture was poured into 20 mL cold water. The formed precipitate was suction filtered and air dried. The reaction products were recrystallized from ethanol.

### 3.2. Procedure B

1 mmol 2,3-dimethylbenzo[d]thiazol-3-ium iodide (**2a**) or 3-heptyl-2-methylbenzo[d]thiazol-3-ium iodide (**2b**) and 1.1 mmol 1-benzyl-4,7-dichloroquinolin-1-ium bromide (**4a**) or 1-benzyl-4-chloro-7-(trifluoromethyl)quinolin-1-ium bromide (**4b**) and 2.2 mmol sodium hydrogen carbonate were ground together in a mortar in the presence of 2 mL of water. The reaction mixture was transferred to a 50 mL Erlenmeyer flask with the help of 2 × 10 mL water and was ultrasonicated vigorously for 20 min. The formed precipitate was suction filtered and air dried. The reaction products were recrystallized from ethanol.

ID1: (Yield A: 64%; yield B: 51%); Mp > 300 °C. ^1^HNMR (DMSO_d6_, δ(ppm)): 4.08 s (3H, H_18_ (NCH_3_)), 5.86 s (2H, H_11_,H_12_ (N^+^CH_2_Ph)), 6.98 s (1H, H_5_ (CH)), 7.32 dd (2H, J = 7.2 Hz, H_14_, H_16_ (CH)), 7.36 dd (1H, J = 7.4 Hz, H_15_ (CH)), 7.41 d (2H, J = 6.90 Hz, H_13_, H_17_ (CH)), 7.42 d (1H, J = 6.70 Hz, H_1_ (CH)), 7.49 dd (1H, J = 8.0 Hz, H_2_ (CH)), 7.70 dd (1H, J = 8.4 Hz, H_3_ (CH)), 7.72 d (1H, J = 9.0 Hz, H_10_ (CH)), 7.87 d (1H, J = 8.3 Hz, H_7_ (CH)), 8.04 s (1H, H_6_ (CH)), 8.11 d (1H, J = 7.7 Hz, H_8_, (CH)), 8.71 d (1H, J = 7.4 Hz, H_4_ (CH)), 8.83 d (1H, J = 9.3 Hz, H_9_ (CH)). ^13^C-NMR DEPT 135 (DMSO_d6_, δ(ppm)): 34.70 CH_3_, 56.98 CH_2_, 89.80 CH, 108.28 CH, 114.04 CH, 118.23 CH, 123.53 CH, 125.53 CH, 127.15 CH, 127.27 CH, 128.53 CH, 128.74 CH, 128.87 CH, 129.56 CH, 145.53 CH.

ID2: (Yield A: 38%; yield B: 32%); Mp > 300 °C. ^1^HNMR (DMSO_d6_, δ(ppm)): 0.81 t (3H, J = 7.1 Hz, H_24_ CH_3_), 1.20–1.32 m (4H, H_23_, H_22,_ CH_2_), 1.30–1.35 m (2H, H_21_ CH_2_), 1.41–1.47 m (2H, H_20_ CH_2_), 1.79–1.83 m (2H, H_19_ CH_2_), 4.72 t (2H, J = 7.3 Hz, H_18_ CH_2_), 5.87 s (2H, H_11_, H_12_ CH_2_Ph), 6.98 s (1H, H_5_ CH), 7.32 d (2H, J = 7.3 Hz, H_13_, H_17_ CH), 7.35 d (1H, J = 7.3 Hz, H_1_ CH), 7.41 dd (2H, J = 7.6 Hz, H_14_, H_16_ CH), 7.46 d (1H, J = 7.3 Hz, H_7_ CH), 7.49 dd (1H, J = 7.9 Hz, H_15_ CH), 7.67 dd (1H, J = 8.4 Hz, H_2_ CH), 7.71 dd (1H, J = 9.1 Hz, H_10_ CH), 7.87 d (1H, J = 8.4 Hz, H_4_ CH), 8.04 s (1H, H_6_ CH), 8.12 d (1H, J = 7.9 Hz, H_7_ CH), 8.73 d (1H, J = 7.4 Hz, H_8_ CH), 8.78 d (1H, J = 9.2 Hz, H_9_ CH). ^13^C-NMR DEPT 135 (DMSO_d6_, δ(ppm)): 14.38 CH_3_, 22.46 CH_2_, 26.41 CH_2_, 27.68 CH_2_, 28.87 CH_2_, 31.61 CH_2_, 39.94 CH_2_, 40.11 CH_2_, 40.27 CH_2_, 40.44 CH_2_, 40.60 CH_2_, 46.49 CH_2_, 56.99 CH_2_, 89.43 CH, 108.41 CH, 114.09 CH, 118.27 CH, 123.63 CH, 125.59 CH, 127.14 CH, 127.35 CH, 128.41 CH, 128.73 CH, 128.95 CH, 129.55 CH, 145.51 CH.

ID3: (Yield A: 61%; yield B: 45%); Mp > 300 °C. ^1^HNMR (DMSO_d6_, δ(ppm)): 4.13 s (3H, H_18_ CH_3_), 5.94 s (2H, H_11_, H_12_ CH_2_Ph), 7.04 s (1H, H_5_ CH), 7.36 d + d (3H, J = 7.3 Hz, H_13_, H_17_, H_2_ CH), 7.40–7.41 m (3H, H_14_, H_15_, H_16_ CH), 7.50 dd (1H, J = 8.1 Hz, H_3_ CH), 7.68 dd (1H, J = 8.4 Hz, H_10_ CH), 7.90 d (1H, J = 8.2 Hz, H_4_ CH), 8.13 d (1H, J = 7.9 Hz, H_7_ CH), 8.21 s (1H, H_6_ CH), 8.79 d (1H, J = 7.4 Hz, H_8_ CH), 9.00 d (1H, J = 8.9 Hz, H_9_ CH). ^13^C-NMR DEPT 135 (DMSO_d6_, δ(ppm)): low solubility in the common solvents.

ID4: (Yield A: 35%; yield B: 58%); Mp > 300 °C. ^1^HNMR (DMSO_d6_, δ(ppm)): 0.80 t (3H, J = 7.0 Hz, H_24_, CH_3_), 1.20–1.25 m (4H, H_22_, H_23_, CH_2_), 1.32–1.36 m (2H, H_21_, CH_2_), 1.42–1.48 (2H, H_20_, CH_2_), 1.79–1.87 m (2H, H_19_, CH_2_), 4.77 t (2H, J = 7.5 Hz, H_18_, N^+^CH_2_), 5.95 s (2H, H_11_, H_12_, CH_2_Ph), 7.06 s (1H, H_5_, CH), 7.33–7.36 m (1H, H_15_, CH), 7.35 d (2H, J = 7.3 Hz, H_13_, H_17_, CH), 7.39–7.42 dd (2H, J = 7.4 Hz, H_14_, H_16_, CH), 7.51 d (1H, J = 7.4 Hz, H_7_, CH), 7.53 dd (1H, J = 7.9 Hz, H_2_, CH), 7.70 dd (1H, J = 8.5 Hz, H_3_, CH), 7.93 d (2H, J = 8.4 Hz, H_10_, CH), 8.17 d (1H, J = 8.0 Hz, H_4_, CH), 8.23 s (1H, H_8_, CH), 8.80 d (1H, J = 7.4 Hz, H_6_, CH), 8.97 d (1H, J = 8.9 Hz, H_9_, CH). ^13^C-NMR DEPT 135 (DMSO_d6_, δ(ppm)): 14.36 CH_3_, 22.46 CH_2_, 26.41 CH_2_, 27.78 CH_2_, 28.86 CH_2_, 28.86 CH_2_, 31.61 CH_2_, 46.70 CH_2_, 57.15 CH_2_, 90.25 CH, 108.79 CH, 114.37 CH, 122.398 CH, 122.42 CH, 123.75 CH, 125.86 CH, 125.86 CH, 127.25 CH, 128.19 CH, 128.80 CH, 129.10 CH, 129.55 CH, 145.94 CH.

## 4. Conclusions

A green chemistry approach is applied for the synthesis of new asymmetric monomethine cyanine dyes. The photophysical properties of the dyes are examined experimentally and theoretically. Theoretical considerations confirm that the shorter wavelength maxima in the absorption spectra of the dyes are due to aggregation and H dimers formation. The most significant fluorescence response upon binding to nucleic acids is observed for ID1—a 183- and 223-fold increase in fluorescence intensity for DNA and RNA complexes. The chlorine-substituted dyes ID1 and ID2 demonstrate slightly higher sensitivity to RNA in comparison to dsDNA. It is demonstrated that elongation of the hydrocarbon chain in the benzothiazole ring substituent leads to a decrease in the fluorescence response of the dye-NA complex. Binding affinity and the mode of interaction of the dyes with NAs are evaluated by fluorescence titration, circular dichroism, molecular docking, and fluorescent intercalator displacement assay. A new reference intercalator for FID assay is proposed.

## Figures and Tables

**Table 1:** Absorption properties of the dyes and their complexes with dsDNA and RNA.

Dye	Buffer	dsDNA λ_abs_		RNA λ_abs_	
	λ_abs_ [nm] ε [L/mol.cm]	λ_1_ [nm] ε [L/mol.cm]	λ_2_ [nm] ε [L/mol.cm]	λ_1_ [nm] ε [L/mol.cm]	λ_2_ [nm] ε [L/mol.cm]
ID1	512 (55 921) 489 (50981)	486 (31 043)	518 (47 917)	486 (35 954)	515 (45 580)
ID2	514 (59 359) 496 (44 737)	492 (44 804)	526 (53 762)	492 (45 403)	523 (41 894)
ID3	518 (45 088) 496 (35454)	489 (31 518)	526 (38 826)	489 (30 344)	522 (40 287)
ID4	520 (43 035) 498 (38 075)	492 (33 651)	529 (38 589)	492 (29 962)	529 (33 020)

**Table 2:** PCM/ HFS/6-31G(d,p) calculated and experimental absorption maxima (nm) for the monomeric dyes and the two most stable π-stacked dimers for each of the dyes.

Dye	Monomer		Dimer	
	λ_calc_ [nm]	λ_exp_ [nm]	λ_calc_ [nm]	λ_exp_ [nm]
ID1	507	512	475 485	489
ID2	502	514	477 487	496
ID3	513	518	479 493	496
ID4	506	520	482 493	498

**Table 3:** Fluorescent properties of the dyes and their complexes with ds-DNA and RNA. I_0_ values refer to the fluorescence of the dyes neat in TE buffer in concentrations given in Figure 3 and Figure S9. The fluorescence of the complexes (I) refers to the highest NA concentration as presented in Figure 3 and Figure S9.

Dye	Buffer λ_(nm)_			Dye + dsDNA λ_(nm)_				Dye + RNA λ_(nm)_			
	λ_ex_	λ_em_	I_0(au)_	λ_ex_	λ_em_	I_(au)_	I/I_0_	λ_ex_	λ_em_	I_(au)_	I/I_0_
ID1	486	631	20.2	486	542	843.1	183.9	486	543	853	223.2
		542	4.6								
ID2	492	641	25.4	492	543.5	219.3	61	492	544.5	216.7	86.8
		544	3.6								
ID3	489	632	7	489	550	221.1	87.1	489	550	152	57.9
		550	1.3								
ID4	492	626	13.5	492	552	22	4.7	492	552	28.2	7.3
		552	4.7								

**Table 4:** Binding constants of the dyes to dsDNA and RNA.

Dye	$\lambda_{ex}$ nm	$\lambda_{em}^{Dye}$ nm	$\lambda_{em}^{Dye-DNA}$ nm	$\lambda_{em}^{Dye-RNA}$ nm	$K_{b}^{DNA}$	$K_{b}^{RNA}$
ID1	486	631	542	543	2.9 × 10^5^	1.3 × 10^5^
ID2	492	641	544	545	5.2 × 10^5^	5.3 × 10^5^
ID3	489	638	550	550	2.7 × 10^5^	2.7 × 10^5^
ID4	492	626	552	552	2.8 × 10^5^	2.2 × 10^5^

**Table 5:** Interaction energies [in kcal/mol] of ID dyes and dsDNA/dsRNA.

Dye	dsDNA		dsRNA	
	In Groove	Intercalator	In Groove	Intercalator
ID1	−11.6	−11.9	−8.5	−9.6
ID2	−13.5	−13.8	−8.2	−10.9
ID3	−11.7	−11.9	−8.4	−10.1
ID4	−12.8	−13.2	−9.7	−11.2

## Data Availability

The data that support the findings of this study are available from the corresponding author upon request.

## Supplementary Materials

The supporting information can be downloaded at https://www.mdpi.com/article/10.3390/molecules29010114/s1: Figure S1. ^1^H-NMR spectra of dye ID1 in DMSO-_d6_; Figure S2. ^13^C-NMR of dye ID1 in DMSO-_d6_; Figure S3. ^1^H-NMR spectra of dye ID2 in DMSO-_d6_; Figure S4. ^13^C-NMR of dye ID2 in DMSO-_d6_; Figure S5. ^1^H-NMR spectra of dye ID3 in DMSO-_d6_; Figure S6. ^1^H-NMR spectra of dye ID4 in DMSO-_d6_; Figure S7. ^13^C-NMR of dye ID4 in DMSO-_d6_; Figure S8. UV/V is absorption spectra of dyes ID3 and ID4 pure in TE buffer and evolution of spectra upon addition of DNA and RNA; Figure S9. Fluorescence spectra of dyes ID3 and ID4 pure in TE buffer and evolution of spectra upon addition of DNA and RNA; Figure S10. UV-VIS absorption spectra of TE buffer solutions of dye ID1 with various concentrations. A calibration curve; Figure S11. UV-VIS absorption spectra of TE buffer solutions of dye ID2 with various concentrations. A calibration curve; Figure S12. UV-VIS absorption spectra of TE buffer solutions of dye ID3 with various concentrations. A calibration curve; Figure S13. UV-VIS absorption spectra of TE buffer solutions of dye ID4 with various concentrations. A calibration curve.
