# Supplementary material for: Asymmetric Monomethine Cyanine Dyes with Hydrophobic Functionalities for Fluorescent Intercalator Displacement Assay
Source: Molecules. 2023 Dec 23;29(1):114. doi: 10.3390/molecules29010114 (PMC10779803; doi:10.3390/molecules29010114)
Supplement: Supplementary file 1 [file molecules-29-00114-s001.zip › molecules-2764789-supplementary.pdf]

Supporting information

Asymmetric Monomethine Cyanine Dyes with Hydrophobic Functionalities for FID Assay

S. Ilieva[1], N. Bozova[1], M. Rangelov[2], N. Todorova[3], A. Vasilev[1, 4]*, D. Cheshmedzhieva[1]*

[1] *Faculty of Chemistry and Pharmacy, Sofia University "St. Kliment Ohridski", 1 J. Bourchier Ave., 1164 Sofia, Bulgaria*
[2] *Institute of Organic Chemistry with Centre of Phytochemistry, Bulgarian Academy of Sciences, 1113, Sofia, Bulgaria*
[3] *Institute of Biodiversity and Ecosystem Research, Bulgarian Academy of Sciences, 1113 Sofia, Bulgaria*
[4] *Institute of Polymers, Bulgarian Academy of Sciences, Akad. G. Bonchev St., bl 103A, 1113 Sofia, Bulgaria.*

Email: dvalentinova@chem.uni-sofia.bg, ohtavv@chem.uni-sofia.bg

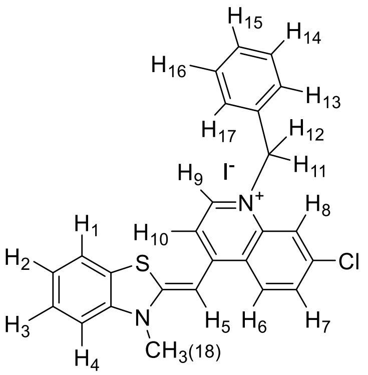

ID1

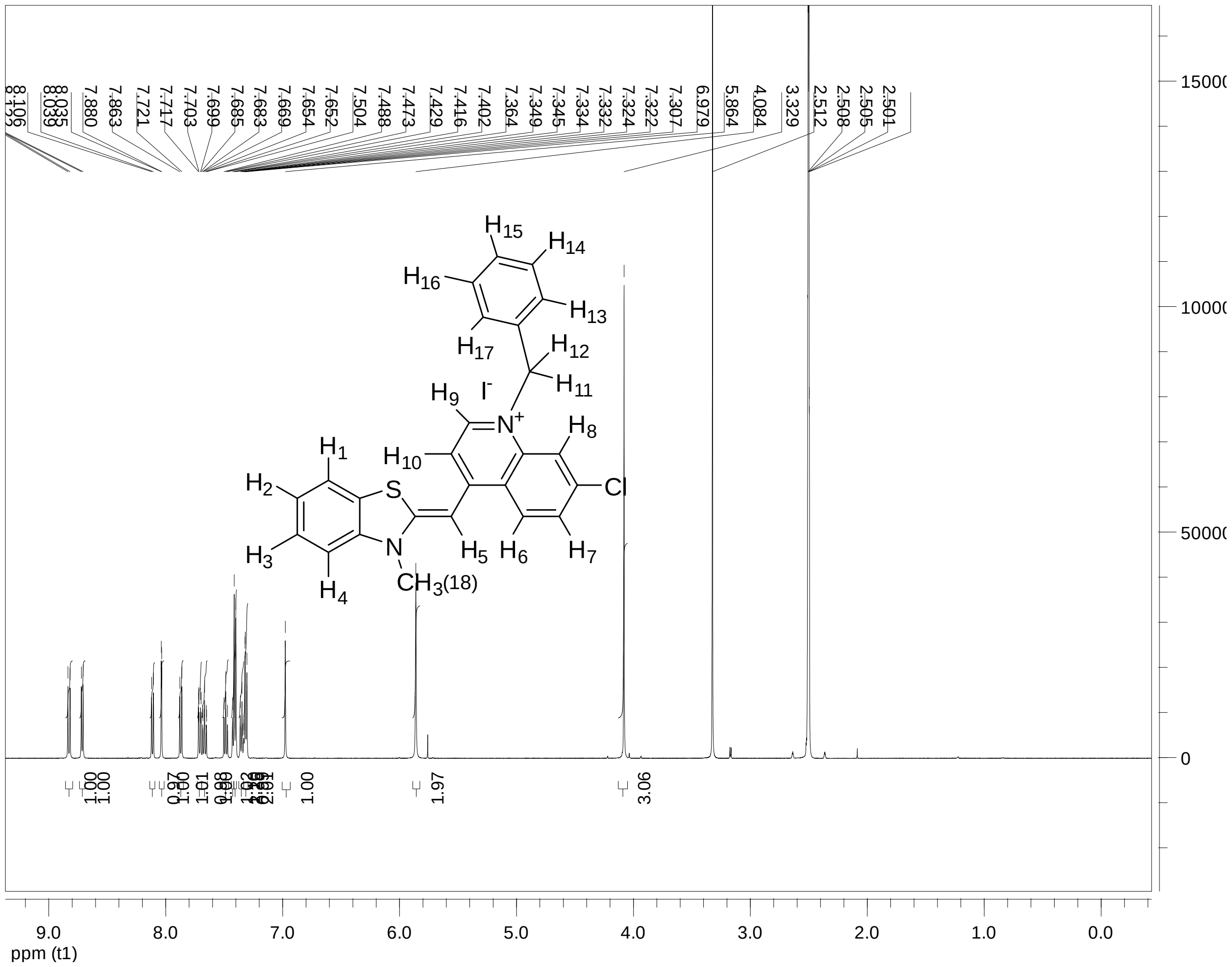

Figure S1. ^{1}H-NMR spectra of dye **ID1** in DMSO-$_{d6}$.

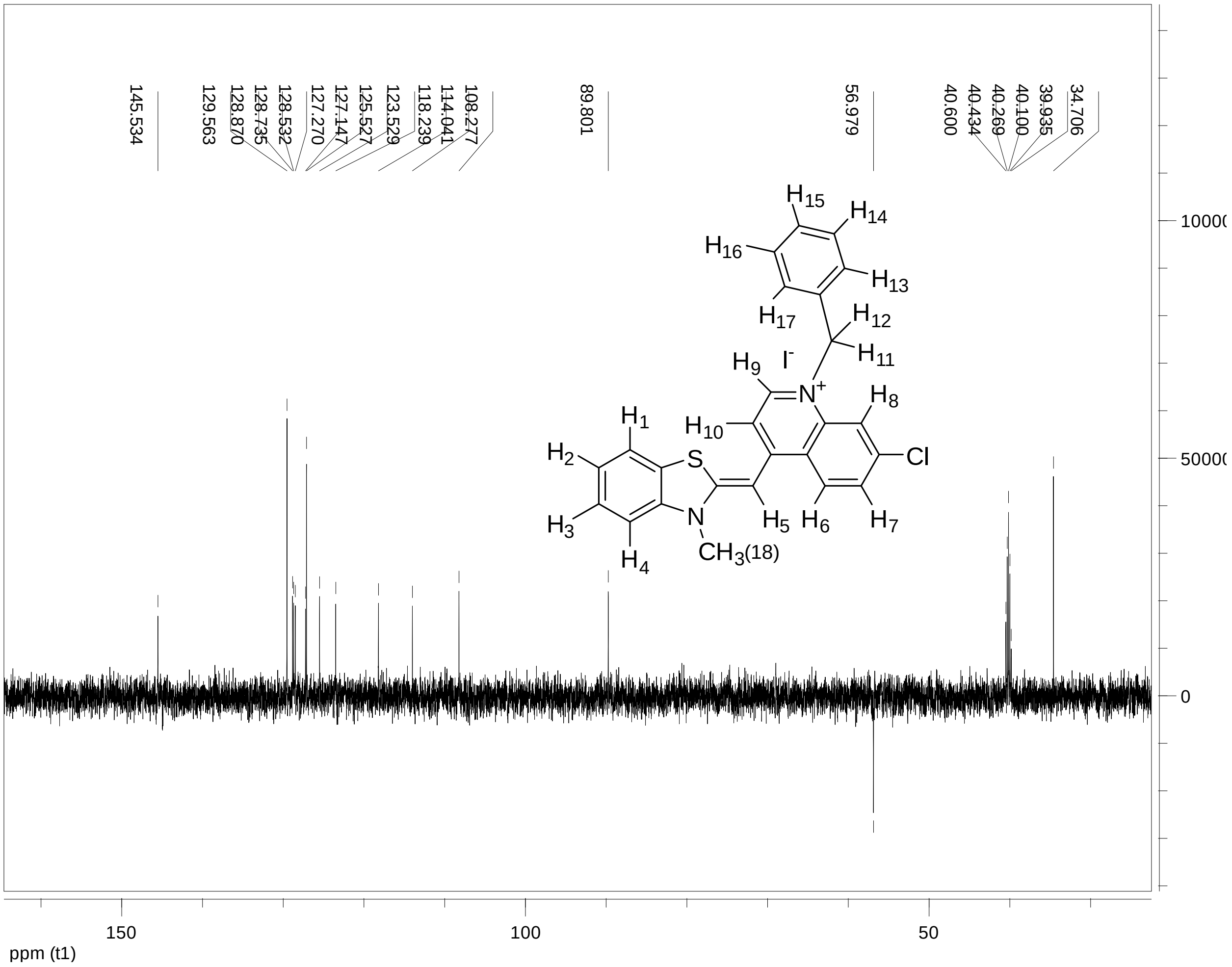

Figure S2. ^{13}C-NMR of dye **ID1** in DMSO-$_{d6}$.

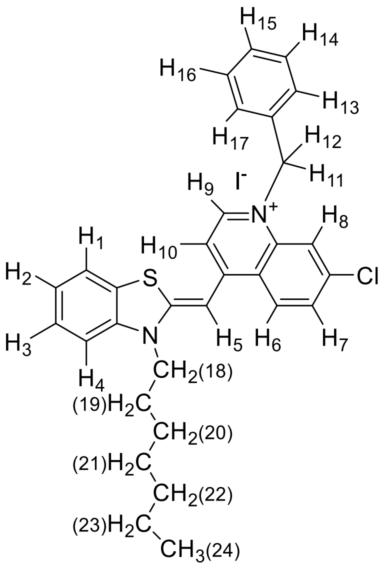

ID2

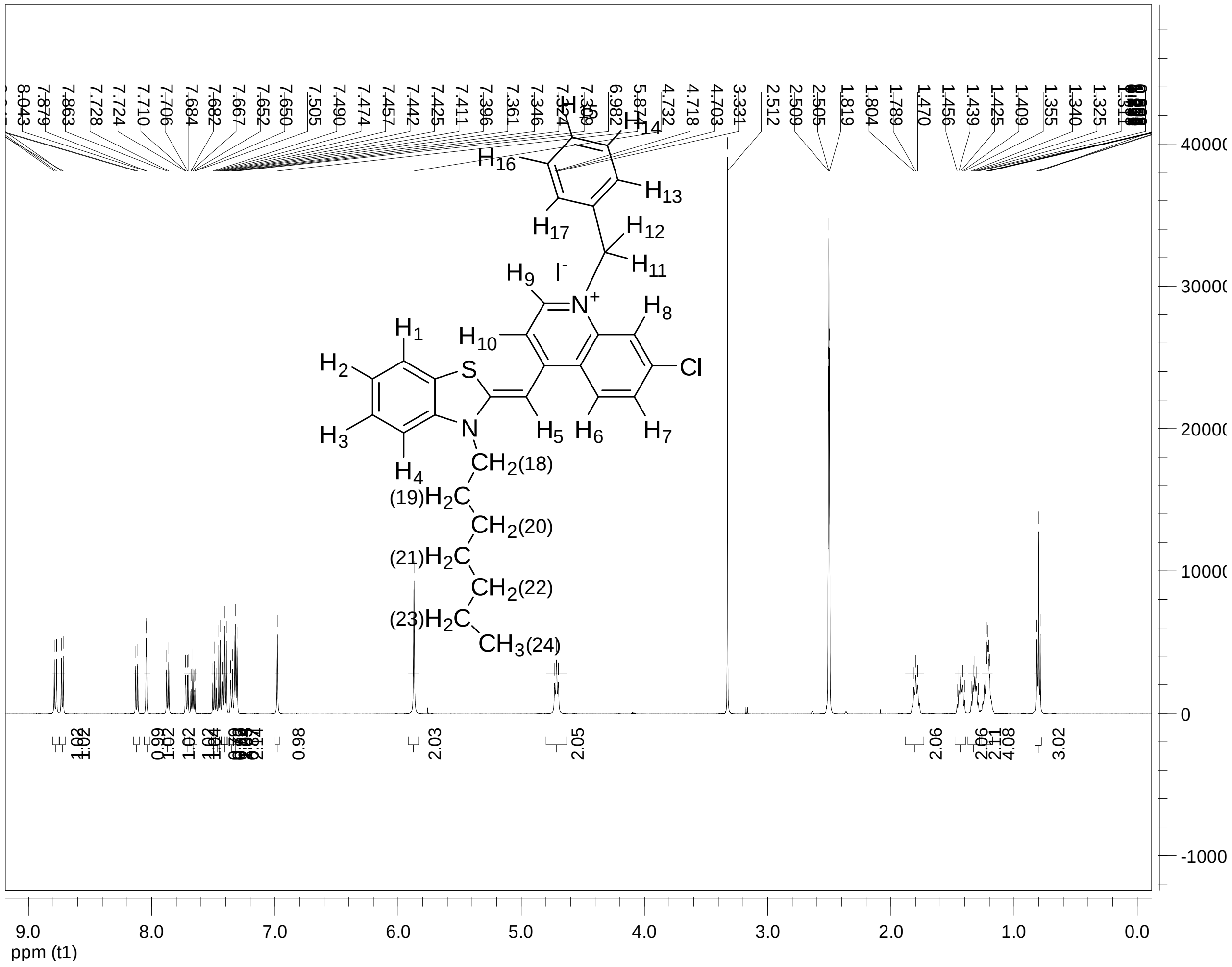

Figure S3. ^{1}H-NMR spectra of dye **ID2**in DMSO-$_{d6}$.

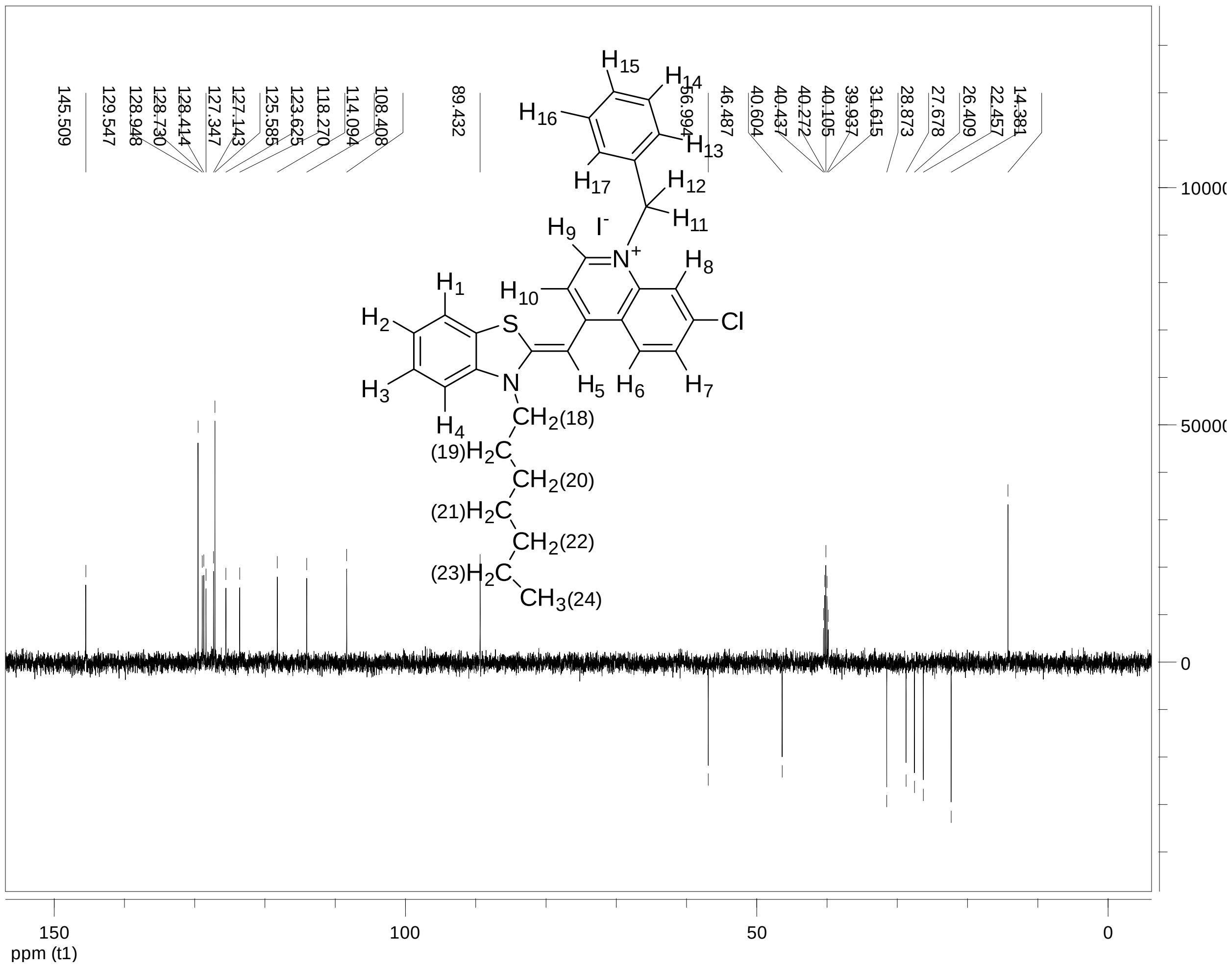

Figure S4. ^{13}C-NMR of dye **ID2** in DMSO-$_{d6}$.

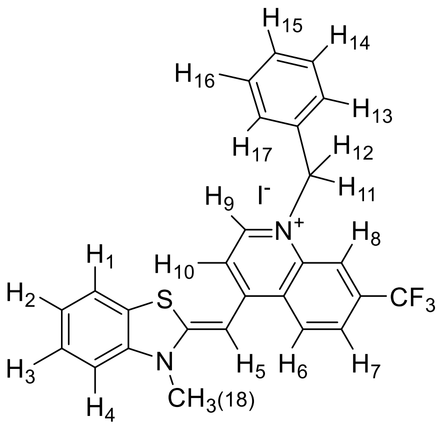

ID3

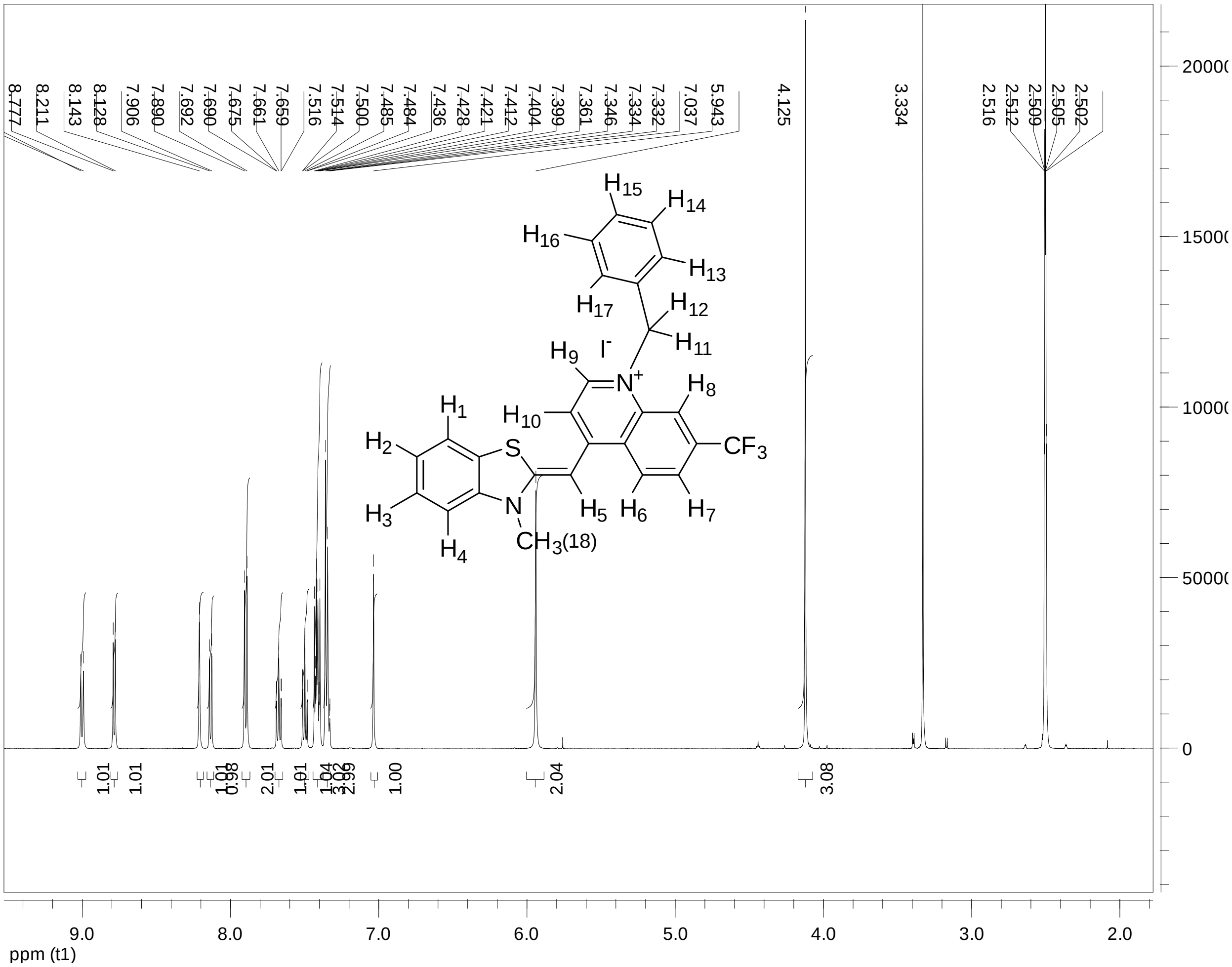

Figure S5. ^{1}H-NMR spectra of dye **ID3** in DMSO-$_{d6}$.

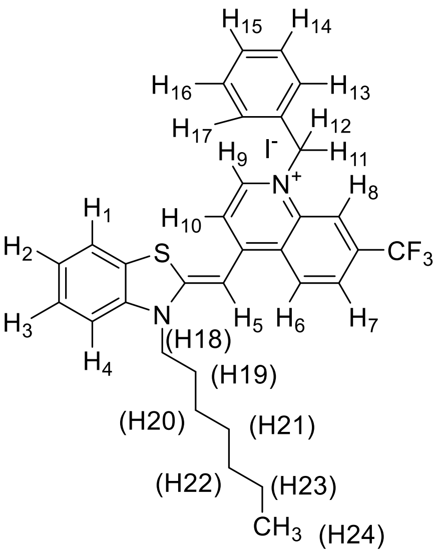

ID4

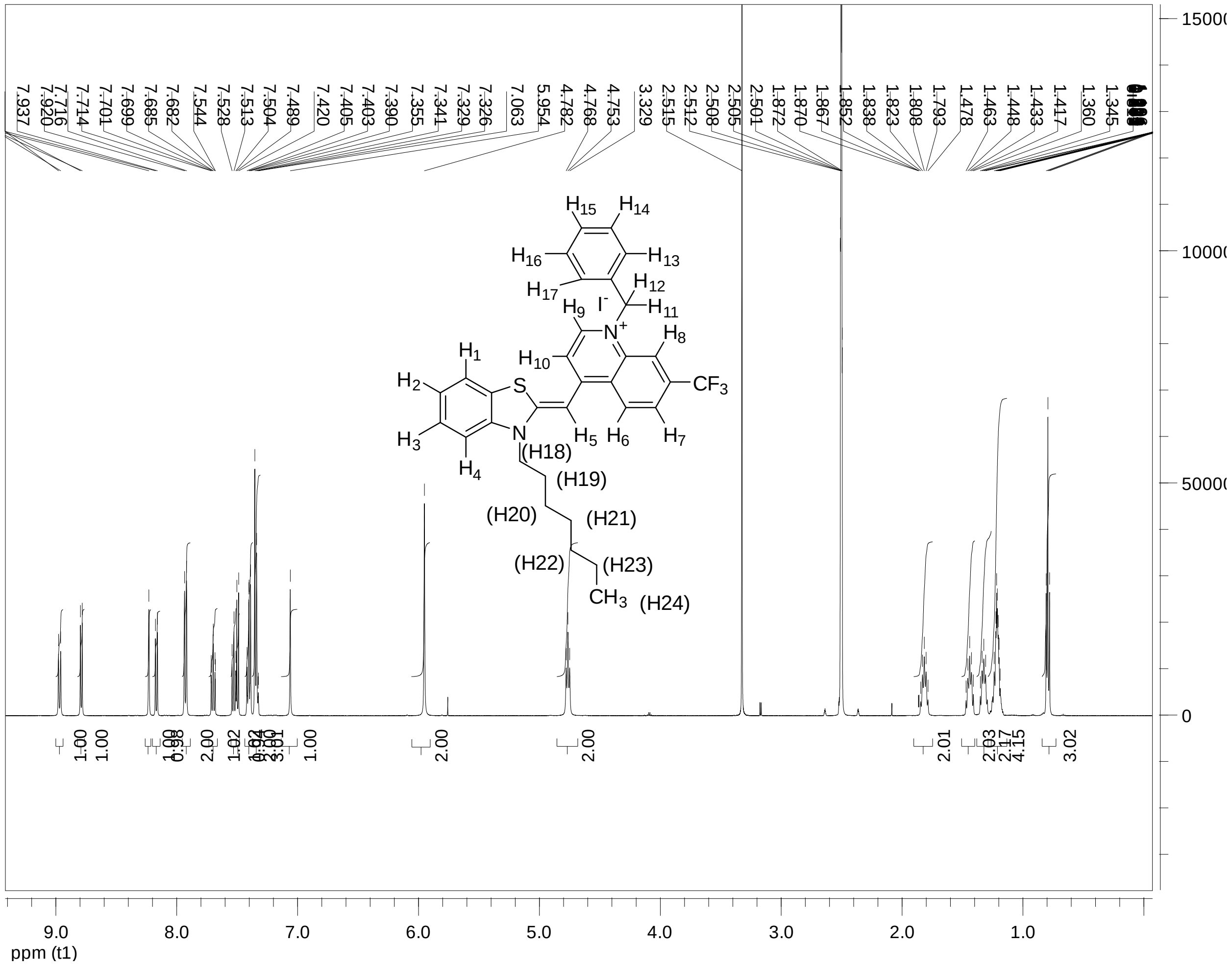

Figure S6. ^{1}H-NMR spectra of dye **ID4** in DMSO-$_{d6}$.

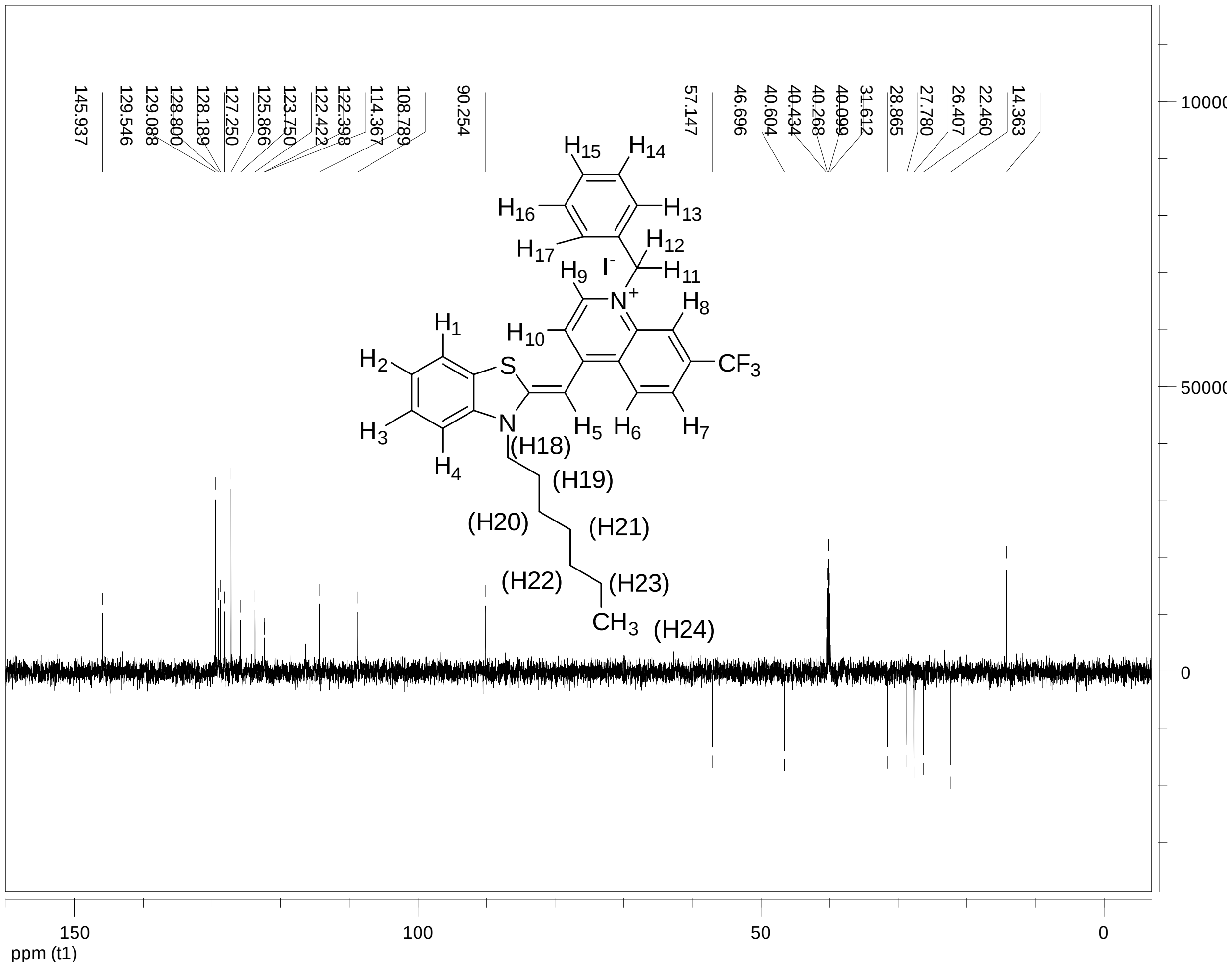

Figure S7. ^{13}C-NMR of dye **ID4** in DMSO-$_{d6}$.

<table>
<tr><th>DNA</th><th>RNA</th></tr>
<tr><td colspan="2">ID3</td></tr>
<tr><td>
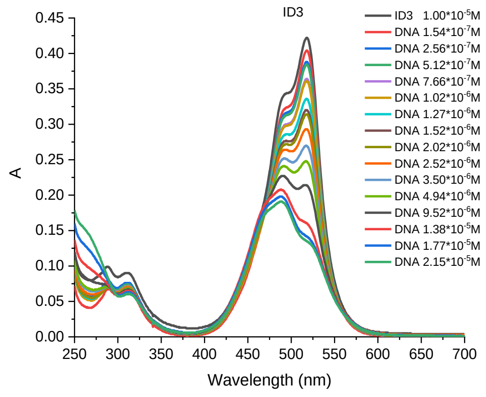

</td><td>
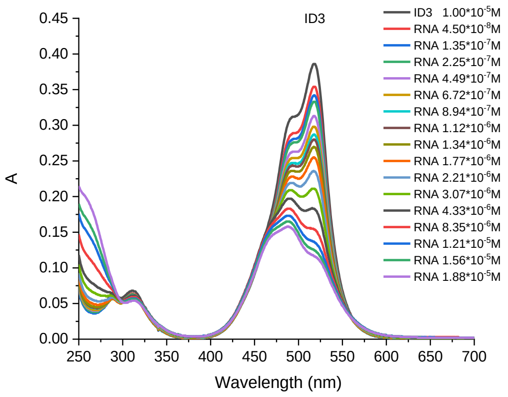

</td></tr>
<tr><td colspan="2">ID4</td></tr>
<tr><td>
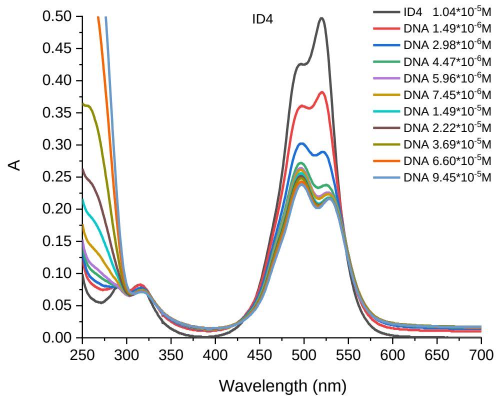

</td><td>
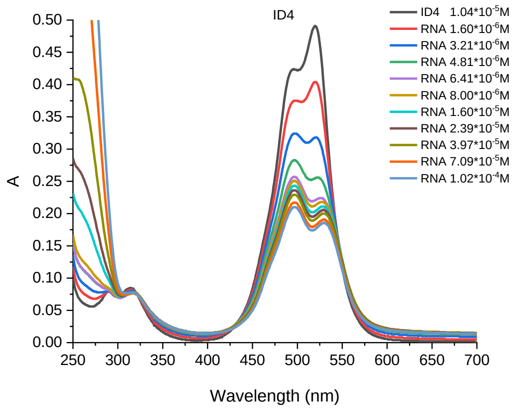

</td></tr>
</table>

Figure S8. UV/Vis absorption spectra of dyes ID3 and ID4 pure in TE buffer and evolution of spectra upon addition of DNA and RNA.

Fluorescence spectrophotometric titrations

<table>
<tr><th>DNA</th><th>RNA</th></tr>
<tr><th colspan="2">ID3</th></tr>
<tr><td>

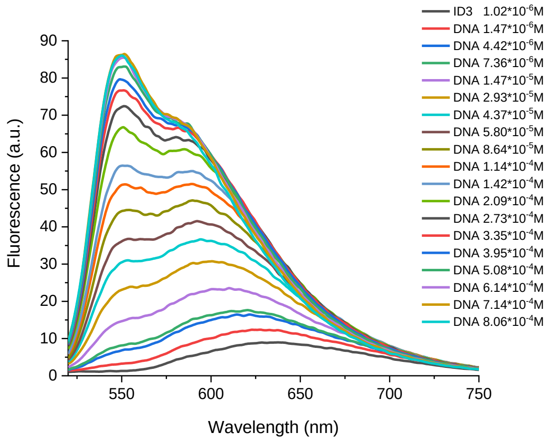

Fluorescence spectrophotometric titrations of ID3 with DNA.

</td><td>

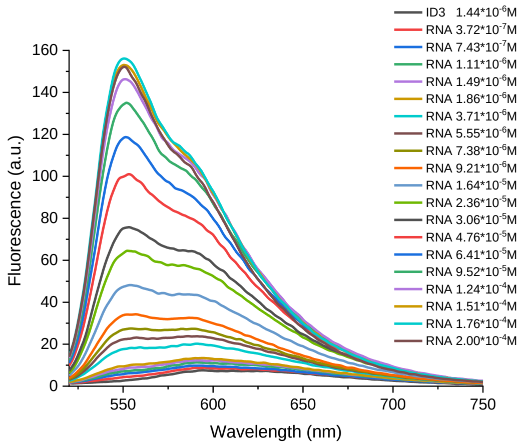

Fluorescence spectrophotometric titrations of ID3 with RNA.

</td></tr>
<tr><td>

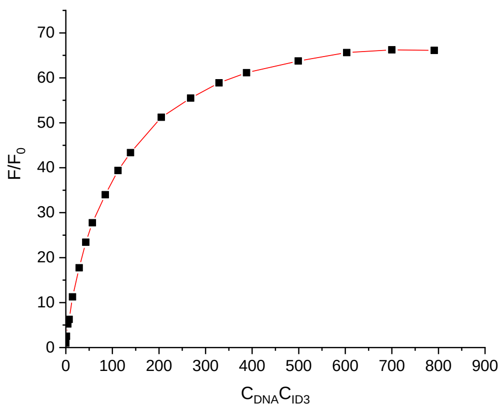

Dependence of ID3 F/F_0 from C_{DNA}/C_{ID3}.

</td><td>

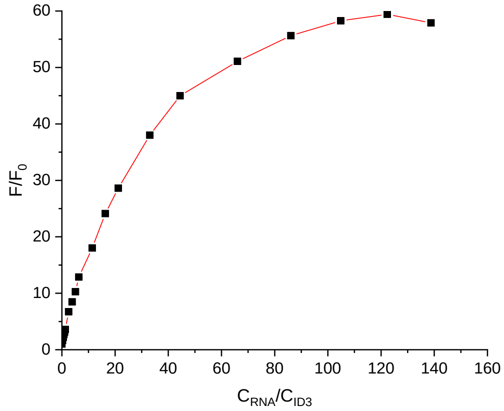

Dependence of ID3 F/F_0 from C_{RNA}/C_{ID3}.

</td></tr>
<tr><td></td><td></td></tr>
</table>

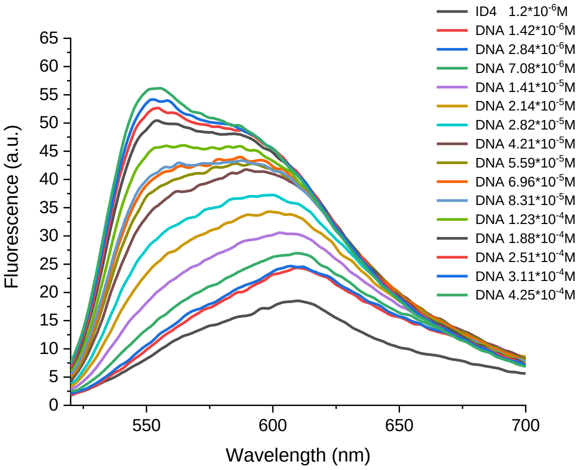

Fluorescence spectrophotometric titrations of ID4 with DNA.

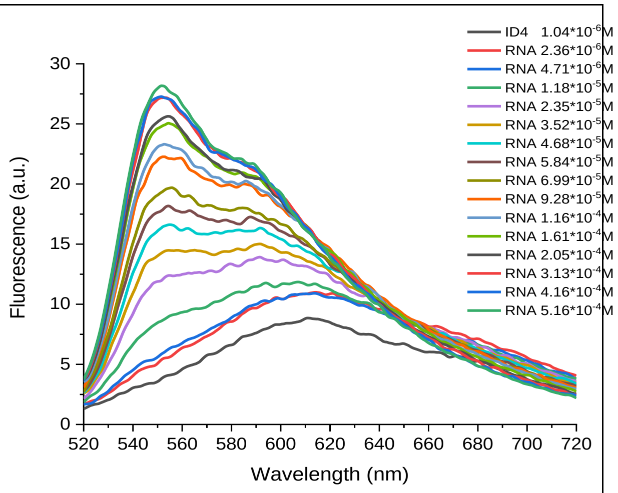

Fluorescence spectrophotometric titrations of ID4 with RNA.

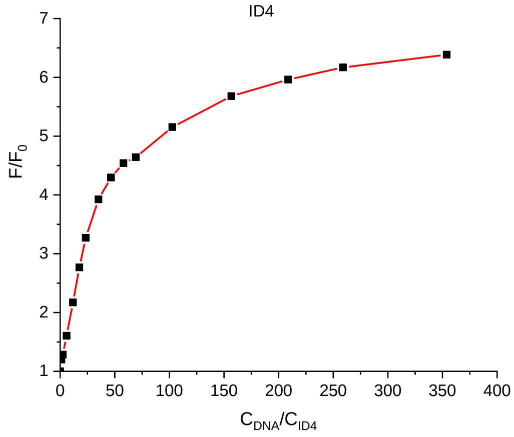

Dependence of ID4 F/F_0 from C_{DNA}/C_{ID4}.

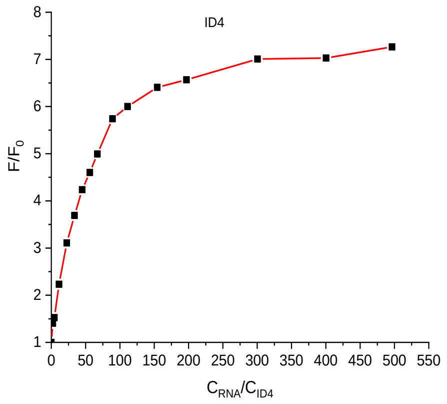

Dependence of ID4 F/F_0 from C_{DNA}/C_{ID4}.

Figure S9. Fluorescence spectra of dyes ID3 and ID4 pure in TE buffer and evolution of spectra upon addition of DNA and RNA.

Dependence of UV-Vis spectra on concentration and calibration curves

ID1

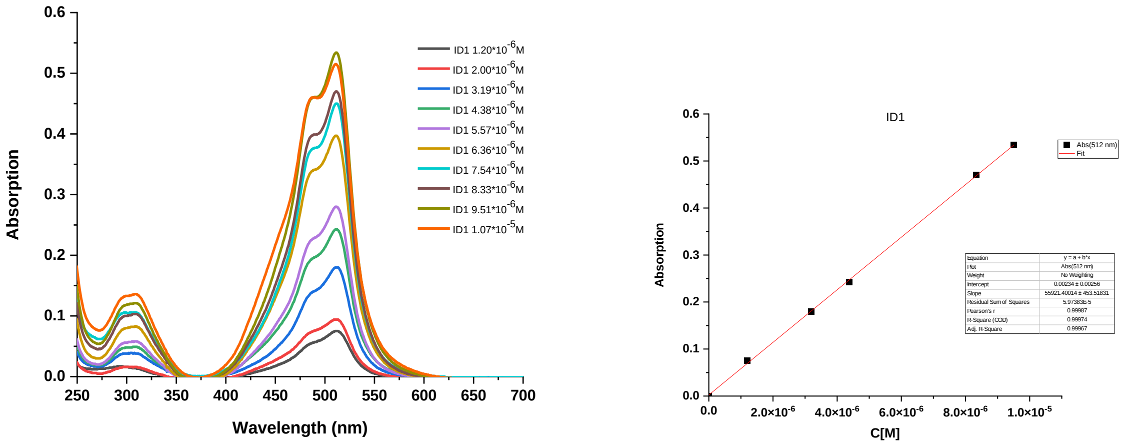

(a) (b)

Figure S10. UV-VIS absorption spectra of TE buffer solutions of dye ID1 with various concentrations. A calibration curve.

ID2

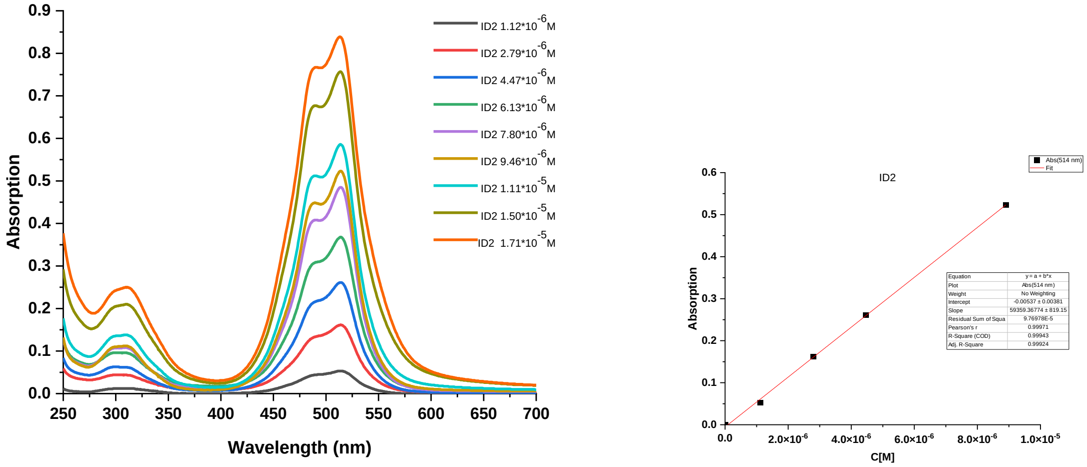

Figure S11. UV-VIS absorption spectra of TE buffer solutions of dye ID2 with various concentrations. A calibration curve.

ID3

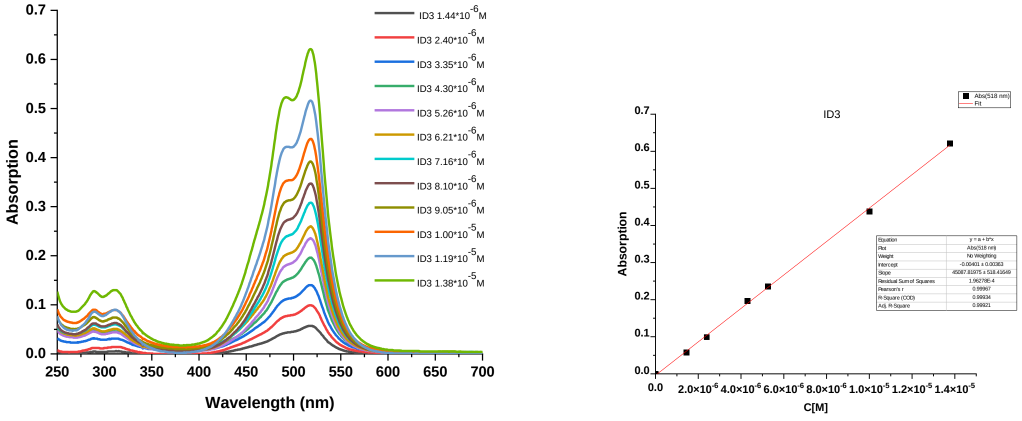

Figure S12. UV-VIS absorption spectra of TE buffer solutions of dye ID3 with various concentrations. A calibration curve.

ID4

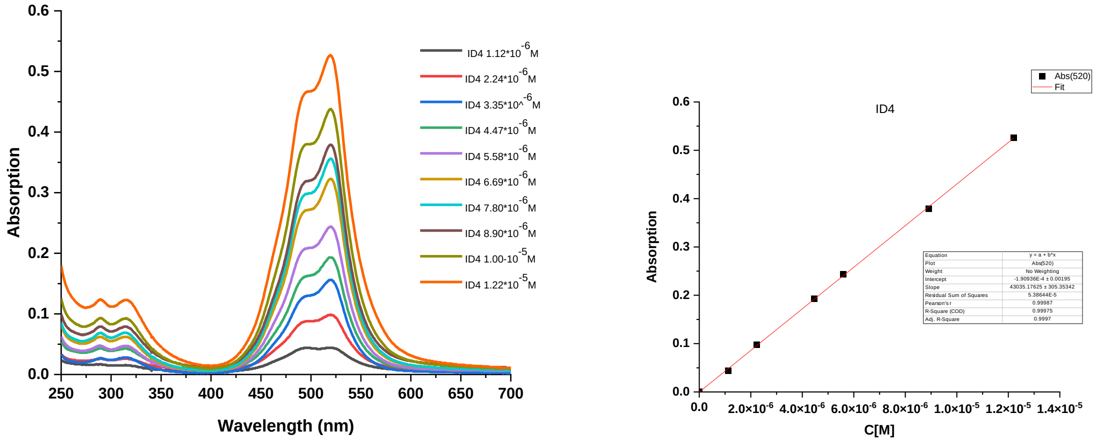

Figure S13. UV-VIS absorption spectra of TE buffer solutions of dye ID4 with various concentrations. A calibration curve.
